# Association of retinitis pigmentosa and advanced keratoconus in siblings

**Published:** 2020

**Authors:** Mihail Zemba, Alexandra-Cătălina Zaharia, Otilia-Maria Dumitrescu

**Affiliations:** * Ophthalmology Department, “Dr. Carol Davila” Central Military Emergency Hospital, Bucharest, Romania

**Keywords:** retinitis pigmentosa, keratoconus, corneal hydrops

## Abstract

**Objective.** The aim of the article was to present the rare association of retinitis pigmentosa and bilateral keratoconus in two brothers, one of whom developed corneal hydrops bilaterally, within a short period of time.

**Methods.** A 29-year-old man presented to our service with corneal hydrops in the right eye, complaining of ocular pain and photophobia. He had a history of retinitis pigmentosa, having been diagnosed as an infant. He also had a younger brother carrying the same diagnosis. Slit lamp examination revealed bilateral keratoconus with corneal hydrops in the right eye, posterior subcapsular cataract, macular atrophy and the characteristic retinal signs of retinitis pigmentosa. The patient’s brother was also examined, with the same findings being noted, apart from the corneal hydrops. We documented the changes using a slit lamp biomicroscope, a fundus camera, a corneal topography, Anterior Segment Optical Coherence Tomography and visual field testing. Right hydrops regressed in one month after hyperosmolar 5% sodium chloride treatment. However, 4 weeks later, the patient presented with the same corneal findings in the left eye. The same treatment was prescribed for the left eye.

**Results.** Corneal hydrops regressed in both eyes with remaining paracentral corneal scars. However, no other treatment for keratoconus was suitable in the case of this patient.

**Discussion:** Retinitis pigmentosa is currently not amenable to any form of treatment, from vitamin supplementation, medical therapy, gene transfer-based therapy, stem cell-based therapy to retinal implantation. However, molecular genetics may someday provide new therapeutic prospects, that could modify the course of RP.

**Conclusions.** The association of retinitis pigmentosa with keratoconus is a fairly rare finding, worth taking into consideration. Also, presentation with keratoconus in such an advanced state is uncommon and, in our case, it was presumably due to the patient’s reduced visual function since childhood, secondary to retinitis pigmentosa, that has prevented him from perceiving any visual modifications caused by keratoconus.

## Introduction

Retinitis pigmentosa (RP) encompasses a group of genetically heterogenous diseases characterized by night blindness and progressive visual dysfunction, secondary to apoptosis of the retinal photoreceptor cells, as well as abnormalities of the retinal pigment epithelium.

Such conditions are determined genetically and can be inherited as autosomal recessive, autosomal dominant or X-linked recessive disorders, but many patients have no family history of the disease or evidence of parental consanguinity. X-linked and autosomal recessive RP tend to have an earlier onset and to be more severe than autosomal-dominant RP [**1**].

The prevalence of RP is approximately 1 per 3000 to 5000 persons, while equally affecting all races and ethnicities, with a slight predominance of the male gender [**2**].

The characteristic fundoscopic features of RP are a pale, waxy optic nerve head, retinal arteriolar attenuation and peripheral retinal pigment epithelial atrophy with bone-spicule pigmentation [**3**]. The disease can be accompanied by complications, such as posterior subcapsular cataract (common and found in all subtypes), open-angle glaucoma, keratoconus (uncommon), posterior vitreous detachment and, occasionally, intermediate uveitis and exudative retinal detachment [**1**].

Keratoconus is a clinical term used to describe an ectatic disease of the cornea, characterized by thinning and protrusion of the cornea, in which it assumes a conical shape. There are many systemic disorders with increased odds of associated keratoconus, such as atopic disease, Down syndrome or connective tissue disorders. Also, keratoconus may appear in the presence of isolated ocular pathology, like Leber congenital amaurosis, retinitis pigmentosa, retinopathy of prematurity or Fuchs corneal dystrophy [**4**].

Over the past 50 years, many authors have reported the association between retinitis pigmentosa and keratoconus, but it remains difficult to establish if retinitis pigmentosa is associated as a cofactor triggering a keratoconus genetic susceptibility or if it is directly involved in the disease pathogenesis.

## Methods and results

**Case presentation**

We presented the cases of two brothers, both diagnosed with retinitis pigmentosa and associated keratoconus. The older brother, a 29-year-old man, came to our Department of Ophthalmology with ocular pain, photophobia, foreign body sensation and epiphora due to acute corneal hydrops in the right eye. The current symptoms had appeared three days before and had worsened over the next days. 

On examination, a hyperemic eye with excessive tearing was noted. Permanent horizontal nystagmus was also present. The patient could follow light, but was uncapable of visual fixation. The best corrected visual acuity was hand motion perception in both eyes.

**Fig. 1:**
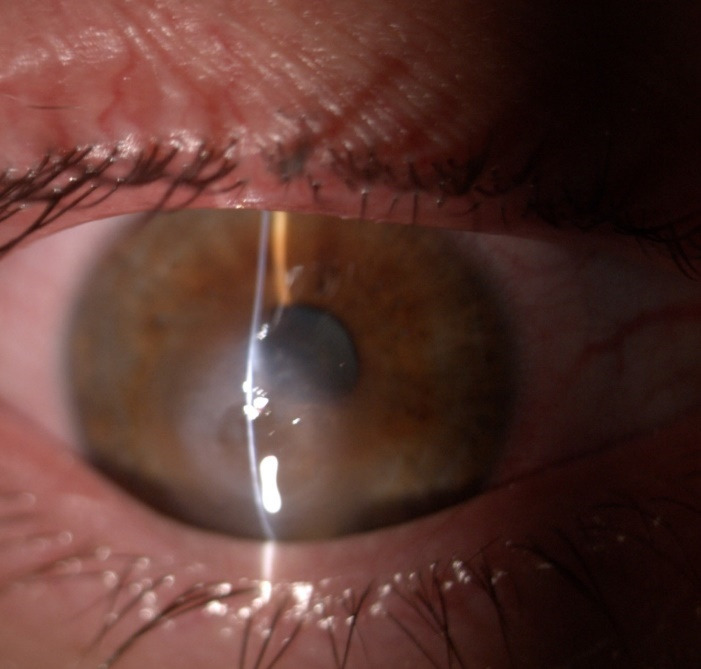
Slit lamp photograph showing acute corneal hydrops of the right eye

**Fig. 2:**
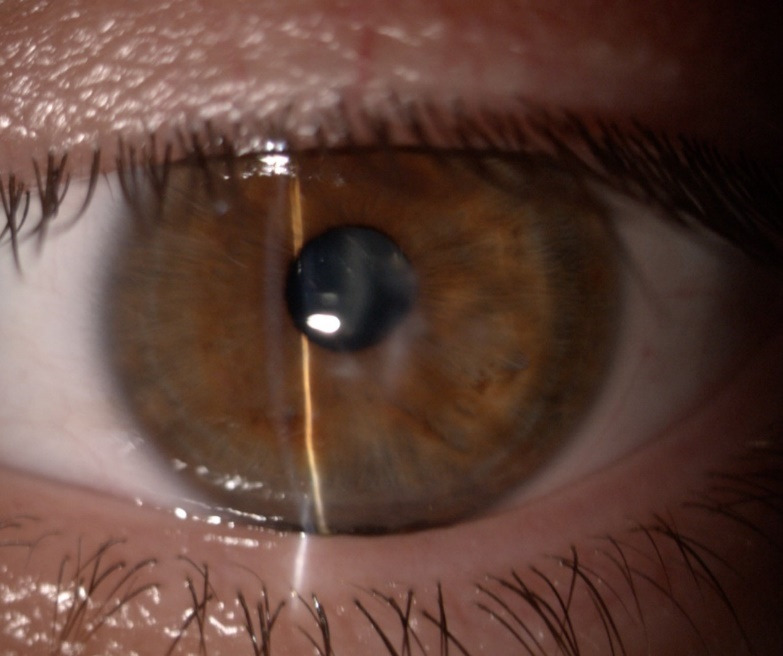
Corneal scarring of the left eye

Photophobia and nystagmus made slit-lamp examination difficult. Corneal findings included keratoconus with inferior paracentral corneal thinning and a positive Munson sign in both eyes and corneal edema with central bulging and epithelial bullae in the right eye (**Fig. 1**). The left cornea showed a paracentral scar, probably the result of previous hydrops (**Fig. 2**). Posterior subcapsular cataract was found in both eyes, a common ocular association in retinitis pigmentosa.

**Fig. 3,4:**
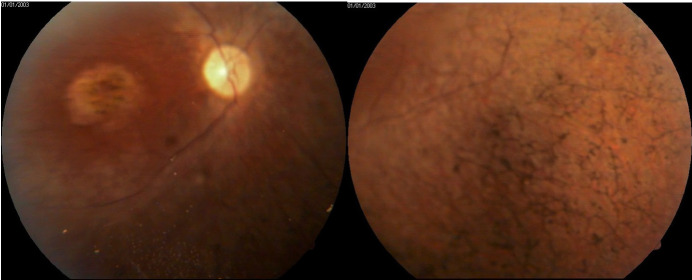
Fundus images showing a pale optic nerve, retinal arterial attenuation, peripheral retinal pigment epithelial atrophy with “bone-spicule” pigmentation and maculopathy

On retinal examination, the clinical triad of retinitis pigmentosa, comprising peripheral “bone spicule” pigmentation, arteriolar narrowing and optic disk pallor was noted bilaterally. Moreover, macular atrophy was present (**Fig. 3,4**).

The patient had been diagnosed in early childhood with retinitis pigmentosa. The patient’s visual acuity, who had never attained 20/ 20, had gradually declined over the years to reach the current best corrected visual acuity of hand motion perception at the age of 13. Family history revealed a brother who had also been diagnosed with retinitis pigmentosa in early childhood, but no other family member with early onset vision loss or who carried a similar diagnosis was identified. 

Visual field testing using the Humphrey Visual Field Analyzer was attempted in order to document the extent of his vision loss for disability purposes, but the patient could not cooperate because of severe field loss. Moreover, as a result of nystagmus and of the inability to maintain focus, cross-sectional images of the central retina by Optical Coherence Tomography, which would have been useful in analyzing the macular changes, could not be obtained.

**Fig. 5:**
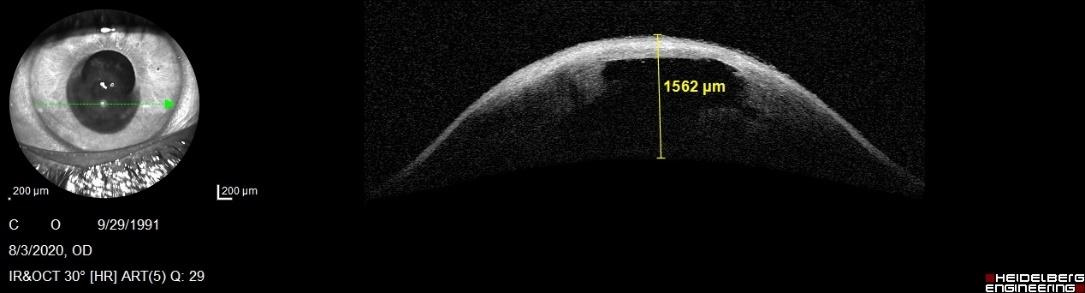
AS-OCT of the right eye showing severe corneal hydrops, with a 1562 μm corneal thickness

**Fig. 6:**
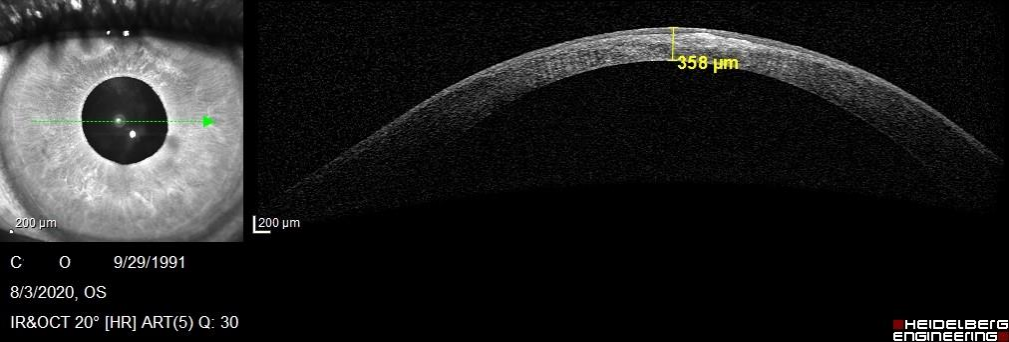
AS-OCT of the left eye showing corneal scarring and a thinner central cornea compared with the right eye

We succeeded to perform an Anterior Segment Optical Coherence Tomography (AS-OCT) to evaluate the morphology of the hydrops. In the right eye, it revealed severe corneal stromal edema with rupture of the Descemet's membrane and a large intrastromal cyst, in the inferior temporal quadrant (**Fig. 5**). Examination of the left eye showed corneal ectasia with apical scarring (**Fig. 6**).

**Fig. 7:**
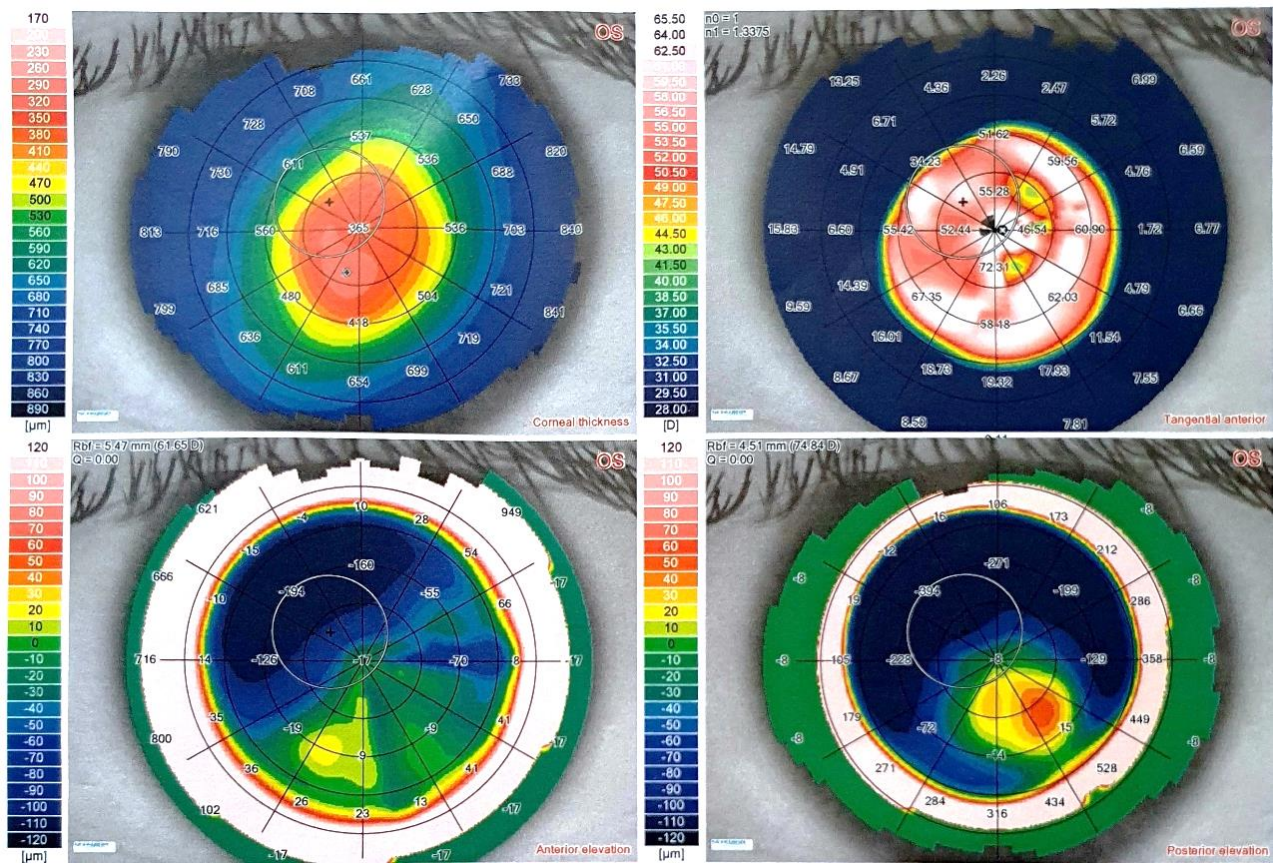
Corneal topography of the older brother’s left eye showing an inferior paracentral thinning of the cornea

Corneal topography of the left eye illustrated prominent inferior paracentral elevation with marked elevation in the posterior elevation map and marked steepening in power map. The pachymetric map showed a minimum corneal thickness point of 310 μm, corresponding to the point of maximal elevation and steepening (**Fig. 7**). 

**Fig. 8,9:**
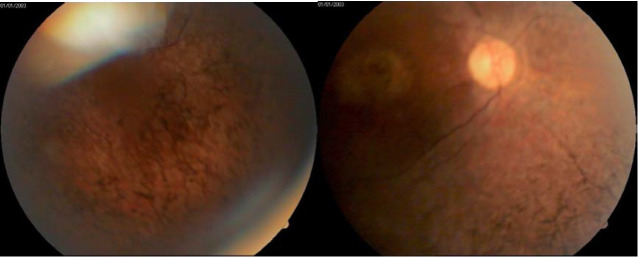
Fundus photographs of the younger brother showing the classical appearance of retinitis pigmentosa

**Fig. 10,11:**
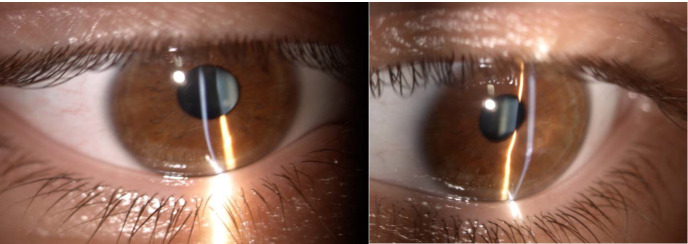
The right and left eye of the younger brother revealing keratoconus and minor inferior corneal scars

Examination of the patient’s 26-year-old younger brother showed the same fundoscopic anomalies, suggestive for retinitis pigmentosa, along with prominent horizontal nystagmus, keratoconus and posterior subcapsular cataract (**Fig. 8,9**). However, keratoconus was mild and only minor inferior stromal scars were found on slit lamp examination (**Fig. 10,11**). The best corrected visual acuity was hand motion perception in both eyes. An attempt was made to obtain a corneal topography and AS-OCT, but results were inconclusive due to a more important nystagmus. 

Genetic testing was recommended, but not performed because of financial limitations.

**Fig. 12,13:**
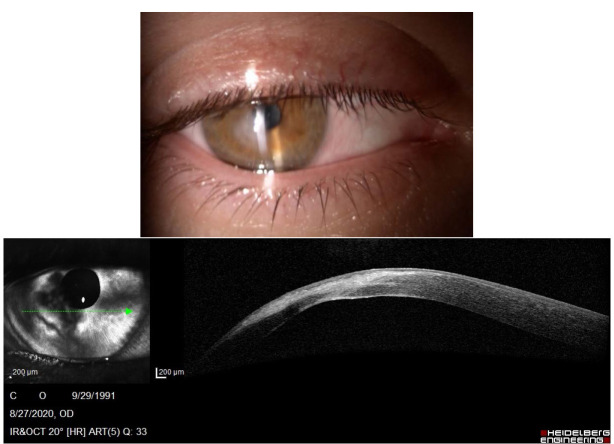
Slit lamp image and corresponding AS-OCT of the right eye of the older brother, showing regression of corneal hydrops in one month and replacement with deep stromal scarring

For the current symptoms, the older brother was treated with a hyperosmotic 5% sodium chloride topical solution applied 4 times a day and a lubricating ointment at night. After one week, the corneal edema had regressed, although not completely, while the photophobia and the tearing had decreased in severity. The treatment was continued for one month (**Fig. 12,13**).

**Fig. 14,15:**
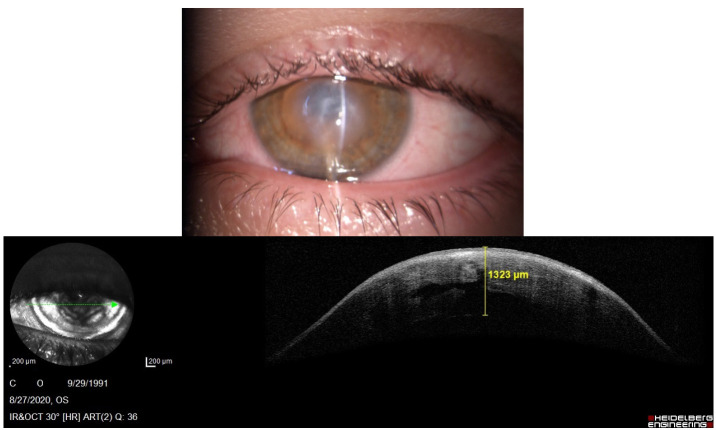
Slit lamp image of the left eye, showing corneal hydrops and corresponding AS-OCT

Four weeks later, the older brother returned with the same initial complaints of photophobia and excessive tearing, this time in the left eye. Clinical examination revealed corneal hydrops with central edema and epithelial bullae in the left eye. The corneal edema of the right eye had been replaced with a white paracentral stromal scar. We performed AS-OCT, which confirmed the diagnosis and was useful in documenting the progression of the corneal disease in both eyes. The same treatment of hyperosmotic eye drops was prescribed (**Fig. 14,15**).

## Discussion

The diagnosis of retinitis pigmentosa was supported by the characteristic fundus findings, which comprise thinning and atrophy of the peripheral retinal pigment epithelium with “bone-spicule” intraneural retinal pigment, gliotic “waxy pallor” of the optic nerve head, and attenuation of retinal arterioles. Another typical feature of RP is the involvement of both eyes, although some degree of difference between the two eyes is expected [**1**].

Moreover, the fundoscopic appearance of our patients revealed bilateral macular atrophy, explaining the reduced functional central vision. The early onset of severe disease, the nystagmus and the atrophic retinal changes imposed a differential diagnosis with Leber’s congenital amaurosis. This condition presents at birth or in the first few months of life with poor vision, nystagmus, abnormal pupillary response and oculo-digital sign, comprising eye pressing and rubbing, likely producing mechanical retinal stimulation, that can result in enophthalmos [**3**].

In addition, another important ocular characteristic association of retinitis pigmentosa - posterior subcapsular cataract, which has 53% prevalence in patients with RP, was identified in our patients [**5**].

The presence of two brothers with the same condition, but no other family member known to be affected, suggests an autosomal recessive inheritance, revealing the genetical determination of RP [**6**]. The patients had been diagnosed with RP in early childhood, but the visual acuity was satisfactory until teenage years, when the central visual acuity was progressively affected. We did not find systemic associations that could be linked with RP in our patients, so we ruled out possible syndromes. 

 The association between pigmentary retinal dystrophies and keratoconus is quoted in the literature as fairly rare [**1**], and retinal dystrophies are not among the most common ocular disorders associated with keratoconus (amongst which vernal conjunctivitis was found to be by far the most common) [**7**]. Nevertheless, attempts have been made over the years to find the missing link that could explain the coexistence of the two in some patients.

In a study conducted by I. Karel in 1968, of the 42 children (22 females and 20 males) with severe retinal dystrophy included, 16 developed keratoconus (5 female and 11 male), in the age group over 15 years, keratoconus being present in 57% of the cases. Of the 16 patients with keratoconus, 7 developed ruptures in the Descemet’s membrane and acute corneal edema. There also seemed to be a link between the severity of the retinal degeneration and the frequency of the keratoconus, the more severe cases appearing to be more prone to developing keratoconus [**8**].

Karel’s study tried to summarize the theories regarding the association of the two entities that had emerged up until the time it was conducted. These theories divided the alleged origin of keratoconus into general and local causes. On the one hand, advocates of the general causes believed that the origin of keratoconus resided in a systemic disorder of mesenchymal tissue, while considering the dysfunction of the diencephalo-hypophyseal system (which is the embryological basis of the retina) as a possible cause. On the other hand, supporters of local causes thought of keratoconus as a consequence of the developmental disturbance and differentiation of the corneal stromal elastic fibers or of the isolated disorder of the Descemet’s membrane and endothelium, with no apparent link with the retinal pathology. Karel also stated that the eye cup, the embryological origin of the retina, could influence the development of the other eye structures in a determining manner and so a disturbance at this level could have repercussions in the corneal development [**8**].

Current directions in the study of the association between pigmentary retinal dystrophies and keratoconus include genetic testing, in an attempt to find common mutations that could link the retinal and corneal abnormalities. 

Sammouh et al. and Bajracharya et al. conducted studies that proposed the possibility of a syndromic association of retinitis pigmentosa, keratoconus and hyperopia, as keratoconus is usually found in conjunction with myopia. However, our patient had an axial length of 22.8 mm, so was neither hyperopic, nor myopic [**9**,**10**]. 

Although the etiology of keratoconus is yet to be uncovered, eye rubbing has emerged as a leading contributor in its pathogenesis [**11**]. It has been observed that patients with significantly impaired vision, especially when of young age, have a tendency to frequently rub their eyes, a phenomenon called oculo-digital reflex. Other diseases in which the oculo-digital reflex is a prominent feature, such as those associated with mental impairment, also exhibit a tendency towards keratoconus formation when compared to the general population. This may suggest that the microtrauma associated with frequent eye rubbing could indeed be the cause for keratoconus formation in these patients, while also possibly explaining the correlation between the severity of the visual impairment and the higher frequency of keratoconus.

Particular in our cases was the presence of keratoconus in both brothers diagnosed in childhood with RP, although the condition was more severe in the older brother, who presented with corneal hydrops and developed bilaterally in the course of one month. Bilateral corneal hydrops is a rare complication of keratoconus, which makes our case distinctive. The delayed diagnosis of keratoconus was due to the late presentation of our patient for medical assessment, because of his inability to identify the initial visual symptoms of keratoconus, such as progressive visual blurring or distortion, which were masked by the reduced visual function caused by retinitis pigmentosa since childhood. 

The management of keratoconus in our patients was difficult for many reasons. The older brother had a severe form, with a minimum corneal thickness point of 310 μm in the left eye, corneal hydrops and central scarring, that could not benefit from the current treatment options, consisting of corneal cross-linking or intrastromal ring segments. Keratoplasty is usually associated with good visual outcome in patients with keratoconus, but our patients had early onset retinitis pigmentosa with severe macular atrophy and poor vision, that would have prevented a significant visual improvement. Even more, the success of corneal transplantation depended on the quality of postoperative care, which is important in preventing and allowing early recognition of complications, such as wound leak, suture-related problems or graft rejection. In our case, the early recognition of any complication by the patients would have been difficult due to their low visual acuity caused by RP [**12**].

Retinitis pigmentosa is currently not amenable to any form of treatment, from vitamin supplementation, medical therapy, gene transfer-based therapy, stem cell-based therapy to retinal implantation [**3**]. However, molecular genetics may someday provide new therapeutic prospects, that could modify the course of RP.

## Conclusions

Retinitis pigmentosa associated with keratoconus is a rare finding that should be taken into consideration. At the same time, the presentation with keratoconus in such an advanced state is uncommon. That is why, in our case, it was presumably due to the patient’s reduced visual function since childhood, secondary to retinitis pigmentosa, that has prevented him from perceiving any visual modifications caused by keratoconus.
